# The Impact of Orthodontic Extrusion on Keratinized Gingiva

**DOI:** 10.3390/medicina60071157

**Published:** 2024-07-17

**Authors:** Ivan Arsić, Nemanja Marinković, Tina Pajević, Jovan Marković, Miroslav Dragović, Zorana Stamenković, Neda Stefanović, Nenad Nedeljković

**Affiliations:** 1 Clinic for Orthodontics, School of Dental Medicine, University of Belgrade, 11000 Belgrade, Serbia; ivan.arsic@stomf.bg.ac.rs (I.A.); tina.pajevic@stomf.bg.ac.rs (T.P.); jovan.markovic@stomf.bg.ac.rs (J.M.); zorana.stamenkovic@stomf.bg.ac.rs (Z.S.); neda.stefanovic@stomf.bg.ac.rs (N.S.); nenad.nedeljkovic@stomf.bg.ac.rs (N.N.); 2 School of Dental Medicine, University of Belgrade, 11000 Belgrade, Serbia; miroslav.dragovic@stomf.bg.ac.rs

**Keywords:** orthodontic extrusion, forced eruption, CBCT, attached gingiva, keratinized gingiva, interdental papilla

## Abstract

*Background and Objectives*: The key factor that enables osteoblastic activity and the formation of new bone, as well as gingiva, during orthodontic tooth extrusion (OE) is the periodontal ligament. The reaction of periodontal tissues associated with changes in the gingiva is a part of orthodontic tooth displacement. The aim of this study was to examine the effect of OE on the width of the zone of the keratinized and attached gingiva, the position of the mucogingival junction, and the height of the interdental papillae in the region where the OE was performed as well as in the adjacent region. *Materials and Methods*: This research included 28 adult patients (both orthodontically treated and untreated). The treated group included 15 patients, in whom orthodontic extrusion of the upper or lower frontal teeth was indicated and performed. The untreated group included 13 patients, with no previous or undergoing orthodontic treatment. Patients with periodontal disease and periodontal pockets in the frontal region and patients allergic to iodine were excluded from the study. Gingivomorphometric measurements were performed on two occasions in three groups of teeth (24 extruded and 30 agonist teeth in the treated patients; 66 teeth in the untreated patients). Statistical analysis of the obtained data was performed using the software package SPSS version 26.0. *Results*: Orthodontic extrusion induced changes in the position of the mucogingival line and an increase in the width of the keratinized gingiva. There were no statistically significant effects on the depth of the gingival sulcus, the attached gingiva width, or the height of the interdental papillae. *Conclusions*: Orthodontic tooth extrusion has an effect on the periodontium in the observed region. Vertical orthodontic force, directed towards the coronal plane, affects the surrounding soft oral tissues.

## 1. Introduction

Orthodontic tooth movement results in dynamic changes in the shape and composition of the bone and soft tissues around the teeth involved in orthodontic therapy. Essentially, the tooth, along with its supporting tissue, moves through the bone, while the alveolar socket migrates [1].

Orthodontic extrusion (OE) involves the movement of teeth using orthodontic appliances in the coronal direction, i.e., in the direction of tooth eruption, which aims to modify the position of the tooth and/or induce changes in the surrounding alveolar and soft tissues [2]. With the use of a straight-wire fixed orthodontic appliance, in the leveling stage of treatment, thin Ni-Ti arch wires produce light, vertically directed forces that provide initial orthodontic extrusive movement [3,4]. OE through this vertical tooth movement stretches the periodontal fibers and induces osteoblastic activity, and, according to some authors, may be used to decrease periodontal pocket depth [3]. The tension created in the periodontal ligament (PDL) by the extrusive force on the tooth leads to bone deposition and coronal migration of the junctional epithelium and induces changes in the width of gingival tissues [5]. The structure and dimensions of keratinized gingiva play a great role in the maintenance of periodontal health [6]. Despite conflicting opinions about the relationship between orthodontic treatments and periodontal health, few studies have addressed the orthodontics-related changes affecting marginal periodontal tissues [7].

The position and dimension of the interdental papillae are very important factors in the esthetics of a smile. It is known that the orthodontic displacement of teeth affects the appearance of the interdental papillae. According to data from the literature, among orthodontically treated adult patients, the incidence of interdental papilla reduction and opening of the triangular space gingivally from the contact of maxillary incisors is about 38% [8]. The same study states that an increased distance from the edge of the alveolar bone to interproximal contact, shorter and more incisively placed interproximal contact, divergence of the crown part of the tooth, and the formation of interdental triangular space are factors that influence the loss of the interdental papilla. At the normal position of the papillae, the roots of the incisors are in a slightly converging position [8]. According to other authors, the interradicular distance and the distance from the contact point/surface to the tip of the alveolar bone septum are crucial for the shape and size of the papilla [9].

Orthodontic extrusion, also known as forced eruption or forced tooth extrusion, can be used to slowly extract teeth that present a poor prognosis [10]. Orthodontic extraction presents a non-invasive, non-surgical therapeutic procedure for enlarging the volume of periodontal tissue that precedes forced “hopeless tooth” loss. It is a safe, highly predictable treatment, and is rarely associated with complications [11,12]. Some authors state that it is necessary to augment soft and hard periodontal tissue before planning a dental implant [13,14,15]. The anatomical features of the gingiva, especially in esthetically and functionally demanding areas, are important in complex implant-retained prosthetic rehabilitations [16]. One of the important aspects of orthodontic treatment is the capability of orthodontics to develop and regenerate osseus and soft tissue [17].

The aim of this study was to examine the effect of OE on the width of the zone of the keratinized and attached gingiva, on the position of the mucogingival junction, and on the height of the interdental papillae in the region where the OE was performed, as well as in the adjacent region.

## 2. Materials and Methods

This study included 28 patients, with their ages ranging from 19 to 30 years, who presented to the Clinic for Orthodontics, School of Dental Medicine, University of Belgrade for orthodontic treatment. Only patients with completed dental and skeletal growth (above 18 years of age) were included in order to avoid the possible passive eruption of teeth that can occur in young patients. There were 15 patients (6 male and 9 female) who underwent orthodontic treatment. The other 13 patients (3 male and 10 female) did not accept orthodontic treatment.

In the treated group of patients, orthodontic extrusion of the incisors or canines was indicated in the upper or lower jaw. Indications for extrusion were a dentoalveolar open bite, vertically displaced canines, and infraposition of the frontal teeth. Extrusion was achieved using fixed orthodontic straight-wire appliances (conventional metal brackets, Roth prescription, slot 0.022”). OE was performed using continuous 0.014” Ni-Ti arch wire in the leveling stage of treatment. Orthodontic extrusion was performed only on one tooth at a time. If there were indications suggesting that OE was needed for more than one tooth in the group of anterior teeth, firstly, initial continuous NiTi 0.014 arch wire was used to engage the vertically displaced tooth with the smallest vertical distance to the occlusal plane. Only one tooth was engaged in order to obtain symmetric alignment with the NiTi wire as much as possible and in order to avoid undesirable tooth movement of the teeth distant to the vertically displaced teeth. When the OE of that single tooth was completed and the leveling phase was finished, stainless steel 0.016 × 0.022” arch wire was engaged with the aligned teeth. In order to prevent arch form distortion and to achieve asymmetric alignment, auxiliary NiTi 0.014 arch wire was placed in the bracket of the next tooth indicated for OE and engaged with the brackets of the other adjacent leveled teeth over the main SS arch wire, with “piggy back” bending of the SS wire in the region of the infrapositioned tooth. These treatment mechanics provided rigid arch wire in the leveled part of the arch, which presented an anchorage for extruding the next tooth in the infraoccluson with auxiliary elastic NiTi arch wire.

The inclusion criteria were good oral hygiene, a healthy periodontium, a thick gingival phenotype with no probe transparency, and the absence of dental plaque and gum bleeding upon probing. Patients who met one of the exclusion criteria (systemic diseases, endodontic treatment of the teeth that were to be extruded, subgingival dental fillings, cigarette smoking habits, poor oral hygiene and periodontal health, iodine allergy due to the use of Lugol solution) were excluded from the study. The total number of tested extruded teeth was 24 and these were labeled as the experimental EVM group. Teeth adjacent to the extruded teeth that were not moved coronally during the orthodontic treatment were also tested (30 teeth in total) and were labeled as the experimental EA group. Since more teeth were extruded in some patients but only incisors and canines were observed, the total number of extruded and adjacent teeth differed between patients.

Patients were scheduled at 4-week intervals for control examinations. Extrusive orthodontic force was measured using a dynamometer and was ≤50 g in all cases. According to some researchers, extrusive orthodontic force should not exceed 50 g per tooth if performed in the frontal region (desirable range: 15 to 50 g) [2,10,18]. In order to avoid complications and undesirable changes in periodontal tissue integrity, the desirable intensity range of OE force was obtained in each patient. The same amount of force was used for incisors and canines.

The amount of extrusion was not more than 1 mm per month.

In the untreated group of patients, we tested 66 teeth in total and those were labeled as the control group.

All patients included in this study signed a written informed consent form for participation that contained basic information about the research.

Two cone-beam computed tomography (CBCT) scans were obtained for each treated patient in the region of the tooth that was indicated for OE (24 teeth in total): one before treatment and one at least six months after OE. CBCT scans were obtained using the Soredex 3D SCANORA system (Soredex, Tuusula, Finland), in high resolution (voxel size 0.1 × 0.1 × 0.1 mm) and the M field of view (80 × 100 mm). The Ethics Committee of the School of Dental Medicine, University of Belgrade approved this prospective study (resolution no. 36/4), which was also performed in accordance with the Declaration of Helsinki Ethical Principles for Medical Research Involving Human Subjects. 

CBCT cross-sectional measurements were performed in the onDemand3D Project Viewer Limited Database, version 1.0.0.1, Cybermed, Seoul, Republic of Korea.

**Figure 1:**
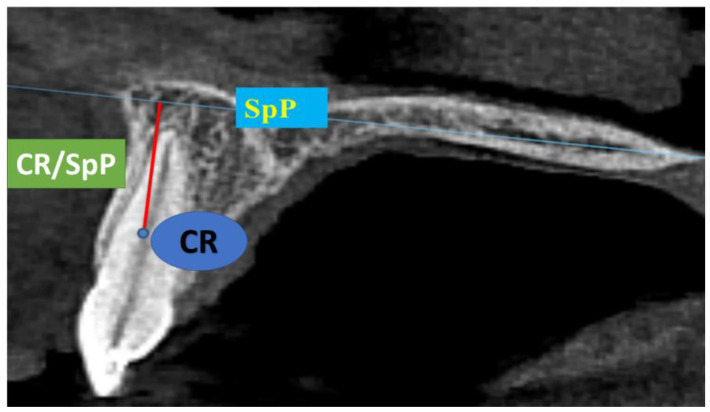
Maxillary plane (SpP), tooth center of resistance (CR), and the distance from SpP to CR on a CBCT sagittal projection.

In the EVM group, CR/SpP and CR/MP presented the shortest distance from the center of resistance (CR) of the tooth to the maxillary plane (SpP, connects the anterior and posterior nasal spines, orthogonal to the sagittal plane) or the mandibular plane (MP, connects the contact point of the mandibular symphysis and the mandibular body anteriorly and the lowest point of the mandibular angle posteriorly) [19]. If the CR/SpP and CR/MP values increased, the extrusion of the tooth was achieved. Therefore, the amount of OE was measured using anatomical structures (SpP and MP) that remain stable in position in non-growing patients. OE was not registered using the position of the neighboring teeth. Vertical movement (VM) of the tooth was presented as the difference between the initial and the final value (∆CR/SpP or ∆CR/MP) [20]. CR was marked as the point on the tooth axis in the sagittal projection between the coronal and the middle third of the root of the tooth, measured from the alveolar crest to the apex of the root (Figure 1) [21].

Gingivomorphometric measurements were performed in the EVM and the EA groups in the region of extruded and adjacent teeth on two occasions: first before the start of orthodontic therapy, and a second time after the removal of the fixed orthodontic appliance (at least 6 months after finishing the OE stage). All of the measurements were made by one examiner. Each parameter was measured three times and the arithmetic mean value was recorded. This was applied for each measurement in both the initial and final recordings.

In the control group patients, these measurements were also performed twice: first when they first presented to the Clinic for Orthodontics, and a second time at least 18 months later in order to match the time intervals between the initial and the final findings in the EVM and the EA groups. Measurements were made in the region of the upper or lower frontal teeth using a ruler with a slide (digital caliper with a measurement accuracy of 0.01 mm) and a graduated periodontal probe with a 0.5 mm section. 

**Figure 2:**
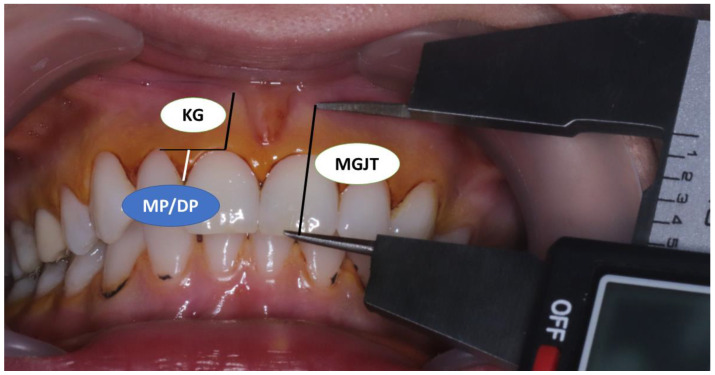
Measurement of soft tissue parameters: distance from mucogingival junction to tooth incisal edge/cusp (MGJT); keratinized gingiva width (KG); mesial and distal papilla height (MP/DP). MGJ was visualized after rinsing with Lugol’s solution.

The following soft tissue parameters were measured:

1. Shortest distance from the tip of the cusp or incisal edge of the tooth to the mucogingival line—MGJT (Figure 2). The mucogingival line was determined via the visual method using 10% Lugol’s iodine solution (Figure 2). Keratinized gingiva does not contain glycogen in the surface layers, so there should be no positive iodine reaction and no change in color [22,23];
2. Shortest distance from the edge of the gingival margin to the mucogingival junction—KG (width of keratinized gingiva) (Figure 2);
3. Papilla height, i.e., the shortest distance from the top of the mesial and distal papillae to the line connecting the marginal gingiva of two adjacent teeth—MP (mesial papillae) and DP (distal papillae) (Figure 2);

4. Depth of the gingival sulcus vestibular in the middle of the mesiodistal diameter of the crown of the tooth—DS;
5. Width of the attached gingival zone—AG, calculated as follows: AG = KG − DS;
6. Change in the position of the mucogingival junction, which was detected on extruded teeth as follows: VM − ∆MGJT < 0—meaning the mucogingival junction moved apically; VM − ∆MGJT = 0—meaning the mucogingival junction did not move; VM − ∆MGJT > 0—meaning the mucogingival junction shifted coronally.

Statistical analysis of the obtained data was performed using the software package SPSS version 26.0 (SPSS Inc., Chicago, IL, USA). The mean, median, and standard deviation were used to describe numerical variables. The One-Sample Kolmogorov–Smirnov test was used to determine the normality of the data distribution, and, in accordance with the results, Kruskal–Wallis and Mann–Whitney tests were used to analyze the differences between the control group and the experimental groups. The results were considered statistically significant if the *p*-value was less than 0.05.

## 3. Results

There were no statistically significant differences between the mean ages of participants in the treated and untreated group (23.78 ± 4.03 and 23.08 ± 2.95 years, respectively). There was no statistically significant difference in the time interval between the initial and final measurements among the untreated and treated groups (4.73 ± 1.99 and 2.45 ± 2.12 years, respectively, *p* ˃ 0.05).

Vertical movement (VM) of the extruded teeth was recorded as the movement of the tooth’s center of resistance towards the coronal plane. This was registered in the EVM group with a value of 1.36 ± 1.02 mm. The maximum VM was 4.50 mm and the minimum was 0.21 mm.

The initial and final findings of soft tissue measurements for the EVM, EA, and control group are presented in Table 1.

**Figure 3:**
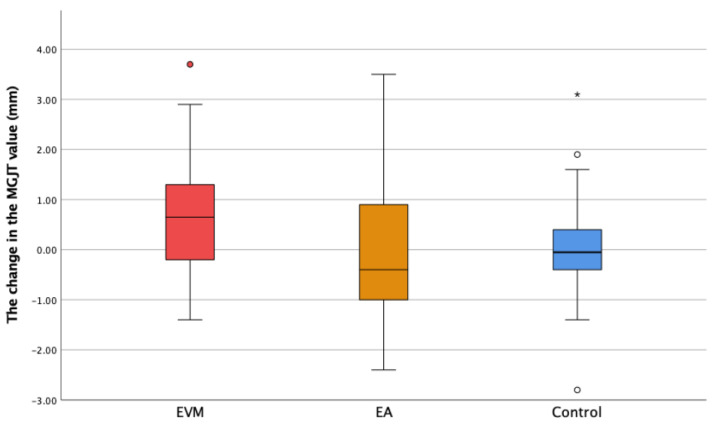
The change in the distance from the mucogingival junction to the tooth incisal edge/cusp (ΔMGJT) in the experimental groups (extruded teeth—EVM; adjacent teeth—EA) and the control group. * Extreme outliers—values that are more than 3.0 × IQR belowQ1 or above Q3 are represented by asterisks. SPSS gives us the case numbers for these values. ° Mild outliers: Values that are more than 1.5 × IQR below Q1 or above Q3 are represented by circles. SPSS gives us the case numbers for these values (white and red circle).

The difference in MGJT between the two measurements (∆MGJT) was significantly larger in the EVM group compared to that in both the EA (*p* = 0.026) and the control group (*p* = 0.012) (Figure 3). 

**Figure 4:**
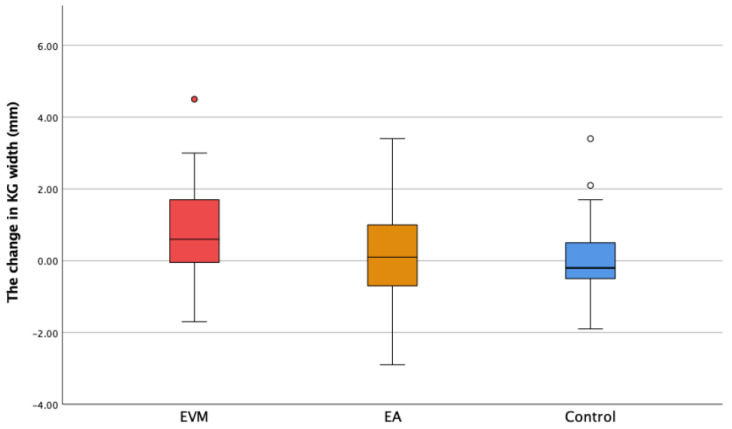
The change in the keratinized gingiva width (ΔKG) in the experimental groups (extruded teeth—EVM; adjacent teeth—EA) and the control group. ° Mild outliers: Values that are more than 1.5 × IQR below Q1 or above Q3 are represented by circles. SPSS gives us the case numbers for these values (white and red circle).

**Figure 5:**
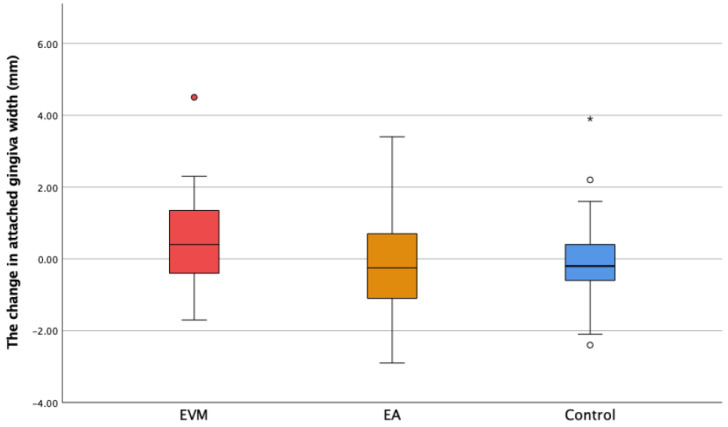
The change in the attached gingiva width (ΔAG) in the experimental groups (extruded teeth—EVM; adjacent teeth—EA) and the control group. * Extreme outliers—values that are more than 3.0 × IQR belowQ1 or above Q3 are represented by asterisks. SPSS gives us the case numbers for these values. ° Mild outliers: Values that are more than 1.5 × IQR below Q1 or above Q3 are represented by circles. SPSS gives us the case numbers for these values (white and red circle).

The initial and final keratinized gingiva width findings showed that participants in this study had a wide zone of KG (more than 2 mm; Table 1). The width of the keratinized gingiva increased with statistical significance in the EVM group compared to the control group (*p* = 0.004). There was no statistically significant difference between EVM and EA, or between EA and the control group (Figure 4). The value of the width of the attached gingiva, calculated by subtracting DS from KG, showed a moderate increase in the EVM group (Figure 5). However, the difference was not statistically different between groups (*p* > 0.05).

**Figure 6:**
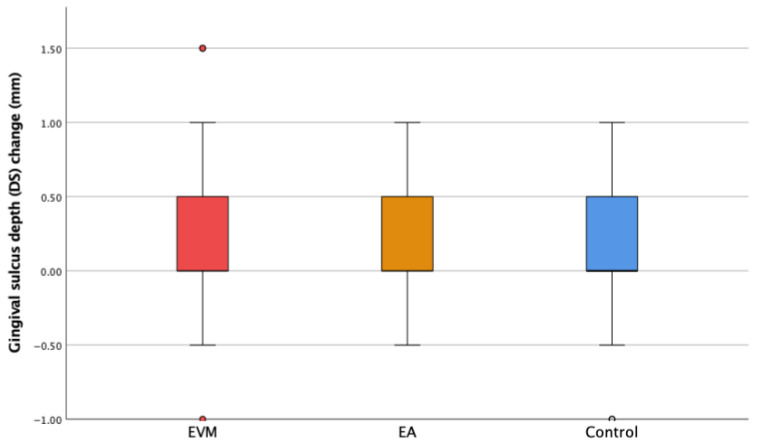
The change in the sulcus depth (ΔDS) in the experimental groups (extruded teeth—EVM; adjacent teeth—EA) and the control group. ° Mild outliers: Values that are more than 1.5 × IQR below Q1 or above Q3 are represented by circles. SPSS gives us the case numbers for these values (white and red circle).

**Figure 7:**
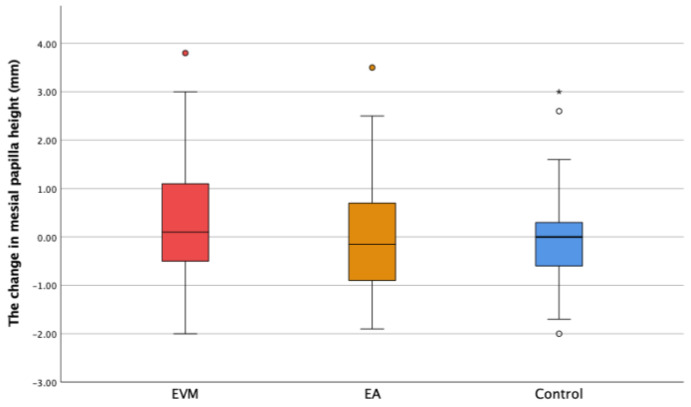
The change in the mesial papilla height (ΔMP) in the experimental groups (extruded teeth—EVM; adjacent teeth—EA) and the control group. * Extreme outliers—values that are more than 3.0 × IQR belowQ1 or above Q3 are represented by asterisks. SPSS gives us the case numbers for these values. ° Mild outliers: Values that are more than 1.5 × IQR below Q1 or above Q3 are represented by circles. SPSS gives us the case numbers for these values (white, yellow and red circle).

**Figure 8:**
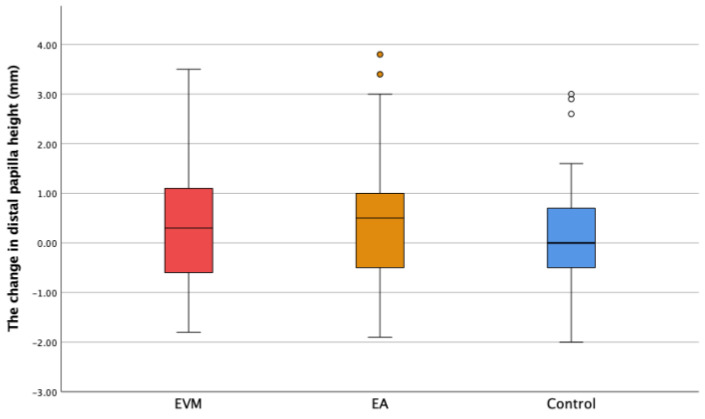
The change in the distal papilla height (ΔDP) in the experimental groups (extruded teeth—EVM; adjacent teeth—EA) and the control group. ° Mild outliers: Values that are more than 1.5 × IQR below Q1 or above Q3 are represented by circles. SPSS gives us the case numbers for these values (yellow and white circle).

The changes in the gingival sulcus depth (ΔDS) and mesial (ΔMP) and distal papilla height (ΔDP) are shown in Figure 6, Figure 7 and Figure 8. Though the depth of the gingival sulcus in the EVM group was slightly increased, there were no statistically significant differences among the EVM, EA, and control groups.

The changes in the mesial and distal papilla height were calculated as the difference between the second and the first measured value. In the EVM group, ∆MP was 0.32 ± 1.45 mm, with a maximum value of 3.8 mm. As for the ∆DP parameter in the EVM group, the change was 0.32 ± 1.30 mm, with the maximum value being 3.5 mm (Figure 7 and Figure 8). In the treated group, no papilla loss was observed, either prior to the OE or after the OE.

There were no statistically significant changes in the mesial and distal papilla height among groups (*p* ˃ 0.05).

In the EVM group, we registered that the MGJ shifted coronally in 50% of the extruded teeth, and apically in 25% of the extruded teeth. In 25% of the extruded teeth, there was no change in the position of the mucogingival junction.

## 4. Discussion

The orthodontic extrusion technique was first described more than 40 years ago. Besides strictly orthodontic implementation, it was introduced as an effective treatment for periodontal infrabony defects [24,25]. Currently, OE is used as a predictable non-surgical procedure for regenerating interdental papillae [26].

In the control and experimental groups, there was an uneven distribution of patients by gender. However, it has been shown that gender does not have a significant influence on the width of the keratinized gingiva [16].

In this research, extruded teeth were allocated to the EVM group and adjacent teeth were allocated to the EA group. This division was made in order to compare soft tissue changes in these two groups, as well as those between these groups and the control group.

The results showed significant change in ∆MGJT in the EVM group. This could be explained by the fact that extruded teeth were moved vertically towards the occlusal plane and there was a slight shift in the position of the mucogingival junction (MGJ). These changes were not registered in the EA group or the control group.

The correlation between the ∆MGJT and VM parameters showed that the change in ∆MGJT value was also caused by the repositioning of the MGJ. Pikdoken et al. reported that the MGJ shifts coronally as a result of OE in 52–55% cases [27], which is in accordance with our results.

The width of the keratinized gingiva (KG) showed an increase in the EVM group. Comparing ∆KG between the EVM group and the control group, there was a statistically significant increase in the EVM group (*p* < 0.05). Comparing this parameter between the EVM and EA groups, the difference was not statistically significant (*p* = 0.076). These results confirm the hypothesis that orthodontic extrusion might increase the width of the keratinized gingiva. Some authors state that the OE of teeth with a poor prognosis as a part of implant site development procedure (ISD) improves the dimensions of the keratinized gingiva [28,29]. More studies can be found that confirm these results [27,30]. Pikdoken et al. investigated the effect of the extrusion of mandibular incisors on the position of the MGJ as well as on keratinized gingiva width. Their results showed that there was MGJ shifting in the coronal direction and improvement of the KG width [27]. However, Alkan et al. observed the effect of sagittal orthodontic tooth movement (protrusive) on keratinized gingiva width and found a significant decrease in the region of maxillary lateral incisors [31].

Similar data can be found in animal studies. During the orthodontic extrusion of teeth, the relationship between the edge of the alveolar bone and the cement enamel junction remains the same [32]. The free gingiva follows tooth movement in the coronal direction to a degree of about 90% of the amount of extrusion, the attached gingiva follows this movement to a degree of about 80% of the amount of extrusion, and the mucogingival junction remains approximately in the same position [33]. In cases where the displacement of the edge of the alveolar bone and the marginal gingiva towards the coronal plane is not desirable (subgingival or infrabony root/crown fractures), authors recommend circumferential supracrestal fiberotomy during OE [34].

A change in the depth of the gingival sulcus (DS) was also registered twice in all three groups of observed teeth. Although depth changes were registered, no statistically significant difference was found between groups. As having a healthy periodontium was one of the basic inclusion criteria for our study, measurements of the depth of the gingival sulcus were performed at a time when there were no signs of gingival inflammation. Inflammation of the marginal gingiva can lead to an increase in the depth of the gingival sulcus. Since inflammation was not reported in the patients included in this study, this could clarify the result of no significant changes in the DS. In the literature, there are data reporting a reduction in sulcus depth after orthodontic tooth extrusion, but within the ISD protocol [28].

The attached gingiva width value (AG) showed a slight increase in the EVM group, but there was no statistically significant difference compared to the EA and control groups. This result is in accordance with the data found in the literature, where some authors stated that OE increases AG and KG width [17,27].

Our study reports slight increases in MP and DP values in the EVM group, but without statistical significance. Bearing in mind that the papilla height was measured from the gingival margin projection of the two adjacent teeth and that the gingival margin followed the extruded tooth movement, it could be said that OE had a positive effect on the papilla dimensions. A recent study that investigated the effect of OE on papilla height reported that OE regenerated missing interdental papillae adjacent to a maxillary anterior single implant [30].

## 5. Conclusions

Orthodontic extrusion of teeth had an effect on the gingival tissue in the observed region. Vertical orthodontic force directed through the longitudinal axis of the tooth towards the coronal plane affected the surrounding soft tissues. It led to an increase in the keratinized gingival zone and a change in the position of the mucogingival junction, most often in the direction of tooth movement. However, these changes were not significant in the region of adjacent unextruded teeth.

According to our findings, OE had no significant effect on the depth of the gingival sulcus, while it caused a slight increase in the width of the zone of attached gingiva and the height of the interdental papillae, albeit without statistical significance.

OE may be taken into consideration as a treatment method for increasing the width of keratinized gingiva.

## Figures and Tables

**Table 1:** Initial and final findings of mucogingival junction to tooth incisal edge/cusp distance (MGJT), keratinized (KG) and attached (AG) gingiva width, gingival sulcus depth (DS), and mesial and distal papilla height (MP and DP) in the experimental groups (EVM—extruded teeth, EA—adjacent teeth) and the control group (mean ± SD).

Group		MGJT (mm)	KG (mm)	AG (mm)	DS (mm)	MP (mm)	DP (mm)
EVM	initial	14.65 ± 1.44	4.83 ± 1.4	3.6 ± 1.35	1.23 ± 0.51	2.23 ± 1.59	2.45 ± 1.25
	final	15.13 ± 1.54	5.44 ± 1.6	3.9 ± 1.47	1.54 ± 0.41	2.67 ± 1.98	2.85 ± 1.63
EA	initial	15.05 ± 1.29	5.41 ± 1.1	4.15 ± 1.06	1.26 ± 0.48	2.34 ± 1.87	2.39 ± 1.36
	final	15.37 ± 1.82	5.78 ± 1.64	4.3 ± 1.52	1.48 ± 0.44	2.28 ± 2.25	3.04 ± 1.79
Control	initial	14.53 ± 1.45	4.99 ± 1.52	3.87 ± 1.49	1.12 ± 0.41	1.5 ± 1.86	2.85 ± 1.63
	final	14.53 ± 1.62	4.99 ± 1.62	3.77 ± 1.41	1.21 ± 0.45	1.47 ± 1.98	2.15 ± 1.44

## Data Availability

Dataset available on request from the authors.
